# Incidence of non-syndromic orofacial cleft during the COVID-19 pandemic in Saudi Arabia

**DOI:** 10.1186/s12889-023-17270-7

**Published:** 2023-12-19

**Authors:** Heba Jafar Sabbagh, Sultan Musaad Alghamdi, Fatma Dawood Abdulhameed, Reema Mahdi Alhussain, Najla Sulaiman Alrejaye, Latifa Yousef AlGudaibi, Bahauddin Ibraheem Sallout, Badi Shoaib Albaqawi, Eman Abdulbaset Alnamnakani, Lougin Khalid Brekeit, Mona Tala Alsharif, Ali Bakr Alshaikh, Manal Ibrahim Almalik, Abdullah Jameel Aburiziza, Norah Suliman Al Soqih, Faisal Ali Alqhtani, Bushra Musaad Alghamdi, Asalah Khalid Alraddadi, Aziza Johar Aljohar, Osama Adel Basri, Rana Abdullah Alamoudi

**Affiliations:** 1 Pediatric Dentistry Department, Faculty of Dentistry, King Abdulaziz University, 21589 Jeddah, Saudi Arabia; 2 Pediatric Dentistry Department, Bisha Dental Centre, Ministry of Health, Bisha, Saudi Arabia; 3 Pediatric Surgery Department, King Salman Medical City, Maternity and Children’s Hospital, Madinah, Saudi Arabia; 4 Pediatric Dentistry Department, Dammam Medical Complex, Eastern health cluster, Eastern Province, Dammam, Saudi Arabia; 5 Orthodontics, King Abdullah International Medical Research CenterConsultant in Craniofacial Orthodontics, Department of Dentistry, King Abdulaziz Medical City, King Saud Bin Abdulaziz University for Health Sciences, College of Dentistry, Ministry of National Guard Health Affairs, Riyadh, Saudi Arabia; 6 Saudi Board of Orthodontics and Dentofacial Orthopedics Program, King Saud Bin Abdulaziz University for Health Sciences, College of Dentistry; King Abdullah International Medical Research Center, King Abdulaziz Medical City, Ministry of National Guard Health Affairs, Riyadh, Saudi Arabia; 7 Obstetrics and Gynecology, Maternal Fetal Medicine, Women’s Specialized Hospital, King Fahad Medical City, Riyadh, Saudi Arabia; 8 Maternal Fetal Medicine, Women’s Specialized Hospital, King Fahad Medical City, Riyadh, Saudi Arabia; 9 Orthodontist, Dental Department, Prince Sultan Military Medical City, Riyadh, Saudi Arabia; 10 Orthodontics and Dentofacial Orthopedics, Prince Sultan Military Medical City, Riyadh, Saudi Arabia; 11 Department of Dental Public Health, King Abdulaziz University, Faculty of Dentistry, Jeddah, Saudi Arabia; 12 Saudi Board of pediatric dentistry, Riyadh Elm University, College of Dentistry, Riyadh, Saudi Arabia; 13 Dental Department, King Fahad Armed Forces Hospital, Jeddah, Saudi Arabia; 14 Pediatric Department, Umm Al-Qura University, Faculty of Medicine, Makkah, Saudi Arabia; 15 Department of Pediatrics, Qassim University, College of Medicine, Buraidah, Saudi Arabia; 16 Pediatric Dentistry Department, King Khalid University, Faculty of Dentistry, Abha, Saudi Arabia; 17 Al-Baha University, Faculty of Medicine, Al-Baha, Saudi Arabia; 18 King Abdulaziz University, Faculty of Dentistry, Jeddah, Saudi Arabia; 19 Department of Dentistry, King Faisal Specialist Hospital and Research Centre, Riyadh, Saudi Arabia; 20 King Faisal Specialist Hospital and Research Centre, Jeddah, Saudi Arabia

**Keywords:** COVID-19, Cleft lip and Cleft palate, Incidence, Prevalence, Saudi Arabia

## Abstract

**Objectives:**

This is the first national study to investigate the incidence of non-syndromic oro-facial clefts (NSOFC) and Pierre-Robin-Sequence in Saudi Arabia over the Covid-19 pandemic period.

**Methods:**

All maternity hospitals (30-hospitals) in the major regions and cities of Saudi from November 2020-to-2021 were included in the study. Patients were evaluated for cleft phenotype using the LASHAL-classification system. The incidence of NSOFC in Saudi Arabia was calculated by comparing the number of NSOFCs cases born out of all live births during the study period at the included hospitals. Clinical examination was performed and information was gathered using a validated data collection form.

**Results:**

In one year, 140,380 live-infants were born at the selected hospitals. Of these, 177 were diagnosed with NSOFC giving an incidence of 1.26/1,000 live-births in Saudi Arabia and the highest incidence in Medina city (2.46/1000 live-births). The incidence of cleft lip-and-palate (0.67/1000 live-births) was higher than that of cleft-palate (0.37/1000 live-births) and cleft-lip (0.22/1000 live-births). Pierre-Robin Sequence incidence was (0.04/1000 live-births). There were 21(12.1) or 23(13.2%) of NSOFC’s mothers exposed or vaccinated with Covid-19, respectively.

**Conclusion:**

The national incidence of NSOFC in Saudi Arabia was 1.26/1000 live births with variation between phenotypes and regions in the country. In addition, to reporting Covid-19 infection prevalence and vaccine exposure among NSOFC's mothers, this study represents the first of its type to evaluate NSOFC prevalence in Saudi Arabia on a national level.

## Introduction

Non-syndromic orofacial clefts (NSOFCs) including cleft lip with or without palate (CL/P) and cleft palate (CP) have various cosmetic and functional complications. These anomalies may cause feeding issues, speech and hearing problems, trouble swallowing, and food regurgitation into the nasal cavity [1]. CP could also occur as a part of Pierre Robin sequence that could cause mechanical obstruction and could result in life-threatening respiratory obstruction and feeding difficulties [2]. Even though these clefts may be repaired surgically in young children, the poor facial development and scarring results in permanent functional and psychological challenges [3]. Children with NSOFCs also have greater rates of morbidity and death than unaffected children [4].

NSOFCs are the most frequent craniofacial abnormality worldwide. They account for 1.47/1000 live births, with significant regional, racial, and rate variations [1, 5].

Saudi Arabia occupies a distinct geographical location between Europe, Africa and Asia and offers promise for research on congenital malformations because of its high birth rate, increased frequency of consanguinity, and mixed racial and cultural backgrounds [6, 7]. Previous research conducted in Saudi Arabia showed vast differences in prevalence ranging from 2.19 to 0.3 /1000 births [8] and were conducted in some cities without national representation [9].

The etiology of NSOFCs is multifaceted and includes genetic and environmental factors and interactions between them [10–12] In addition, the etiology varies regionally and between ethnic groups, making it difficult to explain over the variety of inheritance patterns, distinctions between NSOFC phenotypes, and even the vast range of cultural and geographic variants [1]. Furthermore, the COVID-19 pandemic has caused many psychological and physical trauma. Lockdown, quarantine, and other restrictions on social contact were used to stop the spread of COVID-19, and these measures significantly altered the quality of life for many people. As a result of spending more time at home, people altered their routines and developed new habits, such as starting to smoke again or smoking more cigarettes than before [13]. The adoption of therapies to stop COVID-19 transmission was also expected to have a negative impact on psychosocial family functioning and was associated with greater psychological distress [14]. Expecting mothers worry more about contracting and transmitting the infection. Studies on the COVID-19 pandemic have also shown that psychological discomfort is markedly increased in expecting mothers [15]. Additionally, most Saudi Arabian mothers reported experiencing moderate to severe psychological distress as a result of the COVID-19 epidemic [16]. The changes in lifestyle, stress and infection may influence the incidence of NSOFC.

The aim of this study was to investigate the incidence of non-syndromic oro-facial clefts (NSOFC) and Pierre-Robin-Sequence in Saudi Arabia over the Covid-19 pandemic period. This research represents a prospective analysis of NSOFCs incidence at a national level in all regions and cities of Saudi Arabia for the first time. Additionally, this study is the first of its type to assess NSOFC during the COVID-19 pandemic.

## Materials and methods

### Ethical considerations

The study approval was given by the IRB of King Abdulaziz University (257–07-21), the Ministry of Health (H-02 J-022), King Fahad Medical City (20–642) and King Abdullah International Medical City (H-01-R-005). Parents of affected infants included in the study were informed about the reason, purpose and design of study in both written and verbal form. The parents gave their written consent through an Arabic consent form.

### Design, setting and participants

**Fig. 1:**
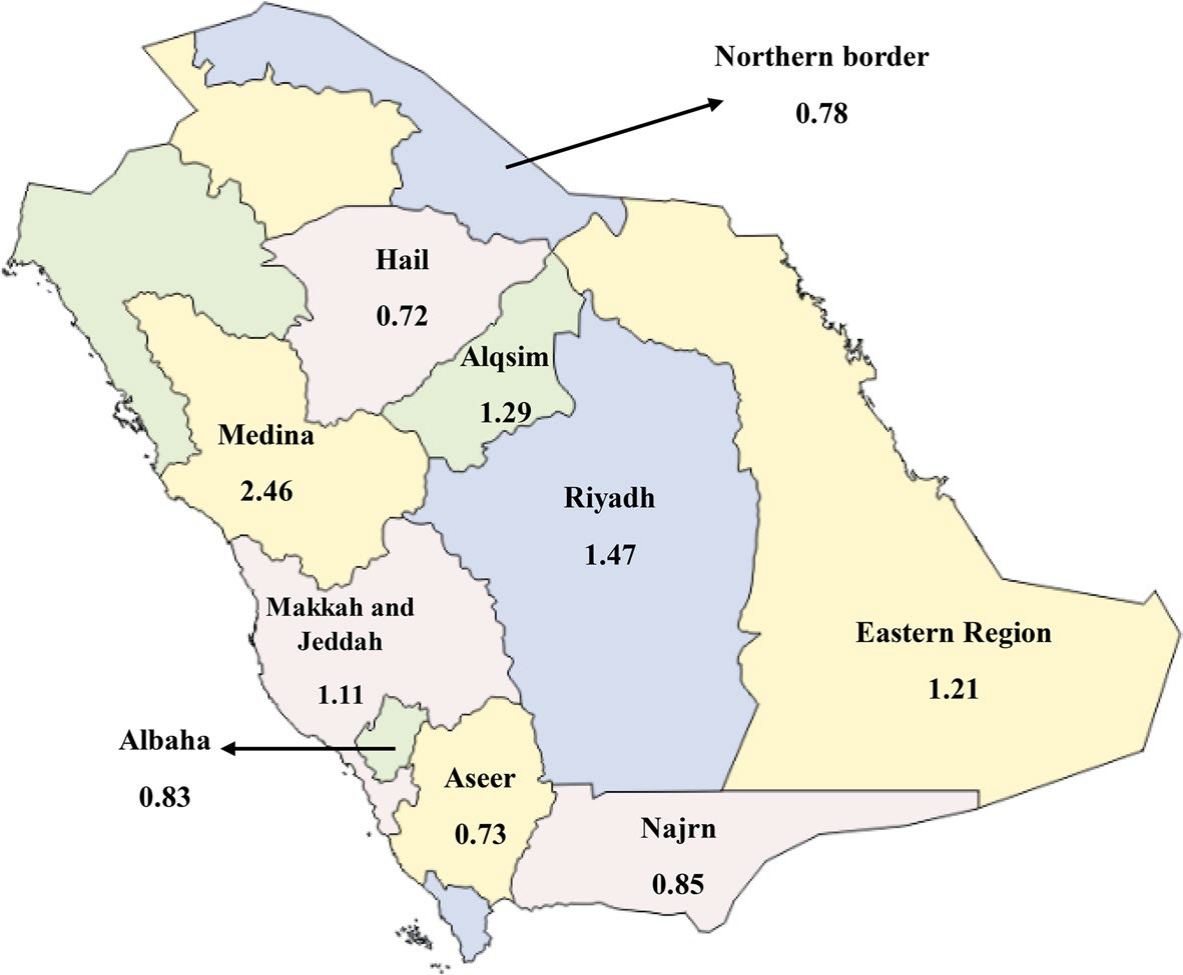
A map of Saudi Arabia showing the NSOFC regional distribution

This prospective study was based on a nationwide population. It assesses NSOFC which is categories in to two main groups: CL/P which include cleft lip only (CL) and cleft lip and palate (CLP); and cleft palate only (CP). The study comprised all government hospitals with maternity units or maternity hospitals (30 institutions) in Saudi Arabia's major regions (see Table 1, Fig. 1). The sampling frame included all live births between November 1, 2020, and November 1, 2021, followed by their immediate screening, isolating NSOFCs afflicted infants, their thorough examination and interviewing their parents. The study duration coincided with the COVID-19 pandemic. Thus, mothers who gave birth during this period had their first trimester during the pandemic. Infants with syndromic OFCs or those who were not born during the study period were excluded.

**Table 1:** List of included region and medical centers

**Region**	**City**	**Hospital**
Central region	Riyadh	King Abdulaziz Medical City (National Guard)
		King Fahad Medical City
		Prince Sultan Military Medical City
		Al-Yamamh Hospital
		King Saud Medical city
	Al-Qassim	Maternity and Children Hospital in Buraydah
		King Saud Hospital in Unaziah
Western region	Makkah	Maternity and Children Hospital in Makkah
		Security Force Hospital
	Jeddah	King Abdulaziz University Hospital
		King Fahad Armed Force Hospital
		King Abdullah Medical Complex
		MOH Hospitals
	Medina	Maternity and Children Hospital in Medina
Eastern region	Eastern province (Ash Sharqiah)	Maternity and Children Hospital in Dammam
		Al-Qatif Central Hospital
		Maternity and Children Hospital in Al-Hasa
North region	Northern border	Maternity and Children Hospital in Arar
	Hail	Maternity and Children Hospital in Hail
South region	Asser	Maternity and Children Hospital in Abha
		Maternity and Children Hospital in Khamis Mushyat
		Mahyal Asser General Hospital
	Najran	Maternity and Children Hospital in Najran
		King Khaled General Hospital
	Al-Baha	King Fahad General Hospital
		Prince Mshari Bin Saud General Hospital

## Methods

Infants with NSOFCs were prospective recruited by nurses present in neonatal wards and intensive care units (ICUs). A network of collaboration and connections was developed over all the hospitals to catch all new births with NSOFCs. After the birth of NSOFC-affected infant, the nurses in each hospital contacted the research coordinator. The research coordinator was in charge of checking the affected infant after an interval of two weeks’ maximum. Furthermore, a retrospective evaluation of the neonatal unit records for all children born with NSOFCs was conducted on a monthly basis to double check births with NSOFCs and all live births and also at the end of the study period to improve ascertainment and precision. Consequently, NSOFC incidence was calculated by dividing the number of infants born with NSOFCs by the total number of live births and multiplying by 1000.

### Clinical examination

To determine the presence of NSOFC and its features, clinical evaluation of affected infants was performed in the pediatric neonatal units and pediatric plastic surgery units using illumination and mirrors. based on the Kriens' categorization. NSOFCs were categorized using the LASHAL classification system; i.e. Lip, Alveolus, Soft Palate, Hard Palate, Alveolus, and Lip [17]. NSOFCs diagnosis and related abnormalities was thoroughly assessed by a geneticist and pediatrician.

The inclusion of NSOFCs and Pierre Robin Sequence and exclusion of syndromic OFCs after examination was based on the geneticist's or pediatrician's diagnosis.

### Data collection

This was carried out prospectively once an infant with NSOFC was born, during their clinical examination. Parents were requested to be available, and they were enquired to sign a consent form. Additionally, they were interrogated to gather information on the newborns' birth, their mothers' exposure to COVID-19 infection and vaccination as follow:

- Sociodemographic data, such as parental age at delivery, grouped in accordance with Herkrath et al. (2012), suggested that the risk of NSOFC arises when fathers reach 40 years of age (< 40 years and ≥ 40 years) and mothers reach 35 years of age (< 35 years ≥ 35 years) [18]; parental educational level (high school and high school or less); and family monthly income, grouped in accordance with Saudi Salary threshold into: low (< 6,000), middle (6,000–16,000) and high (> 16,000) [19].
- Exposure to COVID-19: Cases with positive PCR results have been identified as having Covid-19.
- Exposure to COVID-19 vaccine: For mothers who gave birth after April 25, 2021, when the vaccine was approved by the Saudi Ministry of Health based on the recommendations of a specialized scientific committee, this information was added to the data collection form as soon as the vaccine was made available to the community [20].
- Birth malformations and parental consanguinity run in the family, including first cousins, first cousins once removed, second cousins, and members of the same tribe.

Type of the maternal residency during pregnancy were evaluated. This was preformed Based on population, it is categorized into urban and rural areas. Urban areas are defined as regions having a total population of at least 5,000 individuals, including those that comprise both metropolitan and micropolitan cities, while rural areas are defined as regions with a total population of fewer than 5,000 individuals or outside of the designated urban areas [21].

In order to discuss the study and collaboration, the principal investigators (PI) from each center were acknowledged and brought together. To guarantee ascertainment, the PI provided a thorough description of the study methods in a written document and at face-to-face discussions.

#### Statistical analysis

SPSS Statistics version 20 (SPSS Inc., Chicago, IL, USA) was employed for data entry. Data for the descriptive analysis was gathered using frequencies and percentages. Fisher's exact test and the chi-square test were employed for comparisons. Statistically significant difference was fixed at p < 0.05. The level of confidence and precision of the reported incidence was evaluated with a 95% confidence interval (CI) by means of an online calculator [22].

## Results

### Incidence of NSOFC in Saudi Arabia

At the selected hospitals, 140,380 live children were delivered between November 2020 and November 2021. Out of these, 177 had NSOFC diagnoses, making Saudi Arabia's frequency of NSOFC 1.26 per 1,000 live births. 125/177 (70.6%) CL/P, 52/177 (29.4%) CP, 31/177(17.5%) cleft lip (CL), and 94/177 (53.1%) cleft lip and palate (CLP) were characterized as NSOFC phenotypes. Therefore, for every 1000 live births, 0.89 were CL/P positive. Furthermore, compared to CP (0.37/1000 live births) and CL (0.22/1000 live births), CLP had a greater incidence rate (0.67 for every 1000 live births). The highest incidence of 1.48 for every 1000 live births was found in the western area, followed by the central (1.42/1000 live births), the northern (1.03/1000 live births), and the southern (0.79/1000 live births) regions, as revealed by NSOFC geographical distribution data. Additionally, according to the NSOFC's data based on cities, Medina and Riyadh exhibited greater incidence rates of NSOFC with 2.46/1000 live births and 1.48/1000 live births, respectively. However, Asser and Hail represented lower NSOFC incidence, accounting for 0.72/1000 live births and 0.73/1000 live births, respectively. (Table 2).

**Table 2:** Incidence of NSOFCs from November 2020 to November 2021 according to region and phenotype in Saudi Arabia

**Region**	**City**	**CL (%)**	**CLP (%)**	**CP (%)**	**NSOFC per city/total (%)**	**Total live births**	**Prevalence/1000 live births per city (95% CI)**	**Prevalence/1000 live births per region (95% CI)**
**Central region**	**Riyadh**	11	18	14	43 (24.3)	29,107	1.48 (1.1–2.1)	1.42 (1.1–1.8)
	**Al-Qassim**	4	9	3	16 (9.2)	12,320	1.3 (0.7–2.1)	
**Western region**	**Jeddah**	3	18	7	28 (16.1)	22,177	1.26 (0.8–1.8)	1.48 (1.2–1.9)
	**Medina**	4	16	11	31 (17.8)	12,598	2.46 (1.7 to 3.5)	
	**Makkah**	2	4	2	8 (4.6)	10,366	0.77 (0.3–1.5)	
**Eastern region**	**Dammam**	0	15	6	21 (12.1)	17,254	1.22 (0.8–1.9)	1.21 (0.8–1.9)
**North region**	**N-Border**	4	1	0	5 (2.9)	6,400	0.78 (0.3–1.8)	0.75 (0.3–1.4)
	**Hail**	1	1	2	4 (2.3)	5,529	0.72 (0.2–1.8)	
**Southern Region**	**Asser**	0	7	3	10 (5.7)	13,610	0.73 (0.4–1.3)	0.79 (0.5–1.2)
	**Najran**	2	2	3	7 (4.02)	8,205	0.85 (0.3–1.8)	
	**Al-Baha**	0	3	1	4 (2.3)	4,814	0.83 (0.2–2.1)	
**Total NSOFC phenotype/ total NSOFC (%)** Prevalence/1000 live births (95% CI)		31 (17.5)	94 (53.1)	52 (29.4)	177 (100)	140,380 live births	1.26 /1000 live births (1.1–1.5)	
		0.22 (0.1–0.3)	0.67 (0.5–0.8)	0.37 (0.3–0.5)				

*N-Border* Northern border, *NSOFC* non-syndromic orofacial cleft, *CLP* cleft lip and palate, *CL* cleft lip, *CP* cleft palate

95% CI: The prevalence ratio parameter with 95% confidence lies between the confidence interval limits

### Gender based NSOFC prevalence

The gender-specific distribution of sub-phenotypes of NSOFC in Saudi Arabia is displayed in Table 3. 80 (45.2%) females and 97 (54.8%) males were present in a ratio of 1:1.2. 21 cases of CL were reported in males (67.8%) as compared to 10 cases in females (32.3%), generating 1.9:1 M:F ratio. The distribution of CLP cases among males was also larger than that among females (54 cases, 57.4% vs. 40 cases, 42.5%) giving rise to 1.4:1 M:F ratio. In contrast, there were more CP females than males (31 cases [59.6%] vs. 21 cases [40.4%]), accounting for 1:1.5 M:F ratio. However, statistically significant difference was not present between the two groups (*P* = 0.352).

**Table 3:** Distribution of NSOFC sub-phenotypes born from November 2020 to November 2021 in the Saudi Arabia according to gender

**Phenotype**	**Sub-phenotype**	**Overall prevalence**		
**Cleft lip (CL)** *N* = 31 Male: 21 (67.7%) Female: 10 (32.3%)		Male N (%)	Female N (%)	Total N (%)
	Right complete	1 (1.0)	0 (0.0)	1 (0.6)
	Right incomplete	9 (9.3)	4 (5.0)	13 (7.3)
	Left complete	2 (2.1)	1 (1.3)	3 (1.7)
	Left incomplete	9 (9.3)	5 (6.3)	14 (7.9)
	Bilateral complete	0 (0.0)	0 (0.0)	0 (0.0)
	Bilateral incomplete	0 (0.0)	0 (0.0)	0 (0.0)
**Cleft lip and palate** **(CLP)** *N* = 94 Male: 54 (57.4%) Female: 40 (42.6%)	Right complete	8 (8.2)	5 (6.3)	13 (7.3)
	Right incomplete	12 (12.4)	8 (10.0)	20 (11.3)
	Left complete	8 (8.2)	5 (6.3)	13 (7.3)
	Left incomplete	17 (17.5)	9 (11.3)	26 (14.7)
	Bilateral complete	7 (7.2)	9 (11.3)	16 (9.0)
	Bilateral incomplete	2 (2.1)	4 (5.0)	6 (3.4)
**Cleft palate (CP)** *N* = 52 Male: 22 (42.3%) Female: 30 (57.7%)	Complete	9 (9.3)	15 (18.8)	24 (13.6)
	Incomplete	12 (12.3	16 (20.0)	28 (15.8)
**Total (NSOFC)**		97 (100.0)	80 (100.0)	177 100.0)

*NSOFC* non-syndromic orofacial cleft, *CLP* cleft lip and palate, *CL* cleft lip, *CP* cleft palate

Clefting of the lip among CL/P was more common on the left (17 [9.6%] CL and 39 [22.03%] CLP cases) than on the right (14 [7.9%] CL and 33 [18.6%] CLP cases). This is true both in terms of cleft extension and location. Extension of cleft lip among CL/P, incomplete CL/P was higher (27 [15.2%] CL cases and 46 [26%] CLP cases) as compared to complete CL/P (4 [2.3%] CL and 26 [14.9%] CLP cases). Additionally, the proportion of patients with complete bilateral CLP (16 [9.04%]) vs those with incomplete bilateral CLP (6 [3.4%]) was greater. Additionally, females were more likely to experience complete bilateral CLP (9 [11.3%]) than males (7 [7.2%]), making 1:1.3 M:F ratio. On the other hand, with respect to CP extension, incomplete CP had a greater rate of extension (28 [15.8%]) than complete CP (24 [13.6%]). Also, complete CP occurred more frequently in females (15 [18.8%]) than in males (9 [9.3%]) in a ratio of 1:1.6 (M:F) (Table 3).

### NSOFC associated anomalies prevalence

Table 4 displays the categorization of cases in Saudi Arabia by related abnormalities, gender relationships among them, and NSOFC sub-phenotype. There were 41 (23.2%) cases of NSOFC patients with concomitant abnormalities. Children with CP had a greater prevalence of related malformations (55%) compared to those with CLP (40%) and CL (5%), and these differences were statistically significant (*P* = 0.001). The majority of these abnormalities were congenital heart disease (CHD), limb anomalies (3 [7.3%]), kidney disorders (3 [7.3%]), and face malformations including micrognathia (5 [12.2%]), occurring alone or in combined form in NSOFC children. CHD occurred in NSOFC patients having CLP (13 cases), CP (11, 44% cases) and CL (1, 4% cases).

**Table 4:** Distribution of NSOFC according to associated anomalies and number of children born and their relationship NSOFC sub-phenotype in Saudi Arabia

**Variables**		**Sub-phenotype**			***P* value**	**NSOFC** **N (%)**
		**CL** **N (%)**	**CLP** **N (%)**	**CP** **N (%)**		
**Associated Anomalies**	Yes	2 (4.9)	16 (39)	23 (56.1)	0.001^*∝^	41 (23.2)
	No	29 (21.2)	78 (56.9)	30 (21.9)		136 (76.8)
**Total**		31 (17.5)	94 (53.1)	52 (29.4)		177 (100.0)
**Number of child born**	Twins		5 (4.0)	0 (0.0)	0.171^**¥**^	5 (2.8)
	Singletons		120 (96.0)	52 (100.0)		172 (97.2)
**Total**			125 (53.1)	52 (29.4)		177 (100.0)

*NSOFC* non-syndromic orofacial cleft, *CLP* cleft lip and palate, *CL* cleft lip, *CP* cleft palate

^∝^Chi-square test

^¥^Fissure exact test; significance at 0.05

### Twin pregnancies prevalence

The distribution of cases by twin births is shown in Table 4. There were 5 (2.8%) NSOFC children born as twins. Additionally, there were more cases of CLP (4 [4.3%]) in twin births than CL (1 [3.4%]) cases or CP (0%).

### Pierre Robin Sequence incidence

The study findings indicated that 5 children with PRS were born out of the 140,380 live births that occurred between November 2020 and November 2021. Accordingly, the estimated incidence of Pierre Robin Sequence in Saudi Arabia is 0.04/1000 live births. Out of 5 cases with Pierre Robin Sequence, there were 2 males (40%) and 3 females (60%) with a M:F ratio of 1:1.5.

### Socioeconomic status (SES)

Table 5 displays NSOFC cases distribution based on sociodemographic characteristics. Statistically significant differences were not present between the various sociodemographic attributes and NSOFC phenotypes.

**Table 5:** Distribution of NSOFC according to sociodemographic characteristics and their relationship to NSOFC phenotype in Saudi Arabia

**Variable**		**Sub-phenotype**^**a**^			***P* value**	**NSOFC**^**a**^
		**CL**	**CLP**	**CP**		
**Paternal age**	< 40	23 (79.3)	64 (68.8)	40 (76.9)	0.43^**∝**^	127 (72.9)
	≥ 40	6 (20.7)	29 (31.2)	12 (23.1)		47 (27.1)
**Maternal age**	< 35	21 (72.4)	65 (69.9)	39 (7.50)	0.805^**∝**^	125 (71.8)
	≥ 35	8 (27.6)	28 (30.1)	13 (25.0)		49 (28.2)
**Family income**	< 6,000	12 (42.9)	38 (41.8)	18 (34.6)	0.663^**¥**^	68 (39.6)
	6,000 – 16,000	14 (50.0)	47 (51.9)	27 (51.9)		88 (51.5)
	≥ 16,000	2 (7.1)	6 (13.5)	7 (13.5)		15 (8.8)
**Paternal education level**	< High school				0.096^**∝**^	35 (20.1)
	≥ High school	21 (86.0)	80 (86.0)	52 (100.0)		139 (79.9)
**Maternal education level**	< High school	5 (17.2)	13 (14.0)	11 (21.2)	0.537^**∝**^	125 (71.8)
	≥ High school	24 (82.8)	80 (86.0)	41 (78.8)		49 (28.2)
**Residency description**	Rural	3 (10.3)	10 (10.8)	6 (11.5)	0.984^**¥**^	19 (10.9)
	Urban	26 (89.7)	83 (89.2)	46 (88.5)		155 (89.1)
**Consanguinity**	Yes	12 (41.4)	60 (64.5)	37 (71.2)	0.025^*∝^	109 (62.6)
	No	17 (58.6)	33 (35.6)	15 (28.8)		65 (45.4)
**Family history of NSOFC**	Yes	4 (13.8)	38 (40.9)	18 (34.6)	0.028^*∝^	60 (34.5)
	No	25 (86.2)	55 (59.1)	34 (65.4)		114 (73.6)
**Type of Consanguinity**	1^st^ cousins	11 (61.1)	42 (77.8)	26 (70.3)	0.469^¥^	79 (72.5)
	1^st^ cousins once removed	2 (11.1)	2 (3.7)	1 (2.7)		5 (4.6)
	2^nd^ cousins	2 (11.1)	6 (11.1)	4 (10.8)		12 (11.0)
	Same tribe	3 (16.7)	4 (7.4)	6 (16.2)		13 (11.9)
**Type of consanguinity**	1^st^ cousin	11 (37.9)	42 (45.2)	26 (45.2)	0.577^**∝**^	79 (72.4)
	Other types	18 (62.1)	51 (54.8)	51 (54.8)		30 (27.5)
**Family history of Consanguinity**	Yes	3 (10.3)	19 (20.4)	9 (17.3)	0.724^¥^	31 (17.8)
	No	26 (89.7)	74 (79.6)	43 (82.7)		143 (82.2)

*CL* cleft lip, *CLP* cleft lip with palate, *CP* cleft palate

^**∝**^Chi-square Test

^**¥**^Fisher Exact Test

^a^Number less than the total sample (NSOFC: 174; CL: 31 and CLP: 94) because of missing information

### Consanguinity

Table 5 displays the distribution of cases in Saudi Arabia by consanguinity, family history, and their association with NSOFC phenotype. There were 109 (62.6%) NSOFC children who had consanguineous parents. The proportion of consanguineous parents in CP cases was 71.2%, which was substantially higher than the prevalence in CLP (64.5%) and CL (41.4%) cases (*P* = 0.024).

There were also 60 (34.5%) NSOFC newborns who had a history of the disease in their families. In comparison to CP (34.6%) and CL (13.8%) NSOFC phenotypes, the prevalence of NSOFC family history was statistically significantly greater in CLP neonates (40.9%) (*P* = 0.028).

### COVID-19 infection

The distribution of NSOFC cases regarding absence or presence of maternal COVID-19 infection during first trimester of pregnancy and its association to NSOFC phenotypes in Saudi Arabia are illustrated in Table 6. 21 (12.1%) expecting mothers were exposed to the Covid-19 virus during the first trimester. A statistically significant difference was found between the prevalence of CLP in mothers exposed to Covid-19 infection during the first trimester as compared to CP and CL groups (*P* = 0.024).

**Table 6:** Distribution of cases according to mothers with Covid-19 infection and vaccine during the three-month pre-gestation and 1st trimester periods and their relationship to NSOFC phenotypes in Saudi Arabia

Phenotype	Covid-19 infection			Covid-19 vaccine			Total
	**Yes (%)**	**No** **(%)**		**Yes (%)**	**No (%)**	***P* value**	
**CL**	2 (6.9)	27 (93.1)	0.024^* **¥**^	9 (31)	16 (55.2)	0.021^* **¥**^	29 (100)
**CP**	2 (3.8)	50 (96.2)		5 (9.6)	37 (71.2)		93 (100)
CLP^*^	17 (18.3)	76 (81.7)		9 (9.7)	75 (80.6)		52 (100)
**Total**	21 (12.1)	153 (87.9)		23 (13.2)	128 (73.6)		174 (100)

*CL* cleft lip, *CLP* cleft lip and palate, *CP* cleft palate

^**¥**^Fisher Exact Test

^*^Significance value *P* ≤ 0.05

### COVID-19 vaccination

The findings indicated that 23 (13.2%) of mothers received the COVID-19 vaccine in the first trimester or the pregestation period; 18 (14.8%) of them were those who had CL/P (9 (31%) CL and 9 (9.7%) CLP) and 5 (9.6%) CP children. Compared to CLP and CP, CL mothers received the Covid-19 vaccination at a significantly higher rate (*P* = 0.021).

## Discussion

Over the course of a year, Saudi Arabia had NSOFC birth incidence of 1.26 per 1,000 live births, representing sufficiently lower persistent of disease as compared to its prevalence worldwide (1.47/1000 live births). However, it was considerably higher than the prevalence reported for the country in 2015 (1.17 per 1,000 live births), before the Covid-19 pandemic, and comparable to the cumulative NSOFC prevalence in all studies conducted in Middle East nations as well as Saudi Arabia (1.25/1000 live births) [5, 8, 9].

Only limited research studies have documented the incidence of NSOFC in Saudi Arabia. In these studies, there was either retrospective evaluation of medical records or focusing on a specific hospital setting. Research conducted by Kumar et al. [23] in Riyadh indicated NSOFC prevalence of 0.3/1000 live births, representing lower persistence rate of disease. On the contrary, Borkar et al. [24] found a substantially higher NSOFC prevalence in Al-Qassim (2.19/1000 live births) than global NSOFC prevalence. This investigation was designed to validate these significant discrepancies in above mentioned reports having weak evidences [23, 24]. Another study carried out by Sabbagh et al. [9] assessed NSOFC prevalence in the three Saudi Arabia cities i.e. Riyadh, Jeddah, and Madina, and they found that it was 1.17/1000 live births. The major limitation of this study was the exclusion of other cities of Saudi Arabia.

NSOFC was more incidence in Riyadh (1.48/1000 births) as compared to Jeddah (1.26/1000 live births) and Dammam (1.22/1000 live births). The highest NSOFC incidence, however, was found in Medina (2.46/1000 births). The figure was comparable to research showing that NSOFC incidence was greater in Medina than in Riyadh and Jeddah and higher than the worldwide average [9]. It is noteworthy that Medina reported a greater rate of consanguinity (67.2%) than Riyadh (60%) and Jeddah (44%) [25].

NSOFC incidence in Al-Qassim city (1.29/1000 births) was lesser than the prevalence figure reported in a prior research (2.19/1000 births) [24]. This could be attributed to the excellence of study design as compared to the prior retrospective study, which reviewed patient data but did not perform clinical examination of patients. They also included syndromic cases, and the study wasn't carried out at a maternity hospital. In Narjan, the prevalence of NSOFC was greater than that in a previous research (0.28/1000 births), where it was reported to be 0.85/1000 births [26]. This might be explained by the fact that they did not include all NSOFC patients in their analysis, which was a retrospective examination of medical data. The disparities in NSOFC prevalence rate between cities may also point to various etiological risk factors unique to each region, which would require further analysis by conducting cohort studies for interpretation of accurate and precise results.

In comparison to the global prevalence of CL/P and CP (0.75 and 0.33 per 1000 live births, respectively), this study highlighted greater CL/P and CP prevalence rates (0.89 and 0.37 per 1000 live births, respectively) [27]. However, CP prevalence worldwide (0.33/live 1000) and in Jeddah (0.31/1000 live births) were less as compared to their prevalence in Riyadh (0.48/1000 live births) as well as Medina (0.87/1000 live births) [27]. This may be attributed to the increased occurrence of consanguinity in Riyadh as well as Medina in comparison to Jeddah since consanguinity has greater association with CP than CL/P in earlier research [28].

The reports of our study suggested greater unilateral CL/P prevalence (103/125 cases; 82.4%) than that of bilateral CL/P (22/125 cases; 17.6%). These results concur with those of earlier research as well as global reports [1, 9, 29]. Our results corroborate with those of Mossey et al. [1], who hypothesized that a lower NSOFC prevalence rate would be related with a tendency toward less severe NSOFC. Additionally, our results revealed that the frequency of left-sided unilateral CL/P (56 cases [54.4%]) was greater as compared to that of right-sided (47 cases [46.6%]), taking into account 103 unilateral CL/P cases [1, 9, 29, 30].

In contrast to the prevalences reported by Stoll et al. [31] (35.9%), Fakhim et al. [32] (26%), Aljohar et al. [33] (29.5%) and Pereira et al. [34] (3.2%), the prevalence of related abnormalities was 41 (23.2%) cases, as signified by our results. However, it was comparable to the prevalence rate (20.8%) suggested by Sabbagh et al. in 2015, and the allocation of the cases to the NSOFC phenotypes was the same (17 CLP (23.9%); 16 CP (31.4%) and 9 CL (11.7%)) [31–35]. Finally, our study's prevalence of twins (0.04/1000) was lower than the Saudi population as a whole (4/1000) [36].

### Pierre Robin Sequence incidence

Pierre Robin Sequence is a triad of anomalies that includes micrognathia, cleft palate, and glossoptosis. This sequence could occur as non-syndromic, or could occur associated with other syndromes [2]. In this study we measured the prevalence of the non-syndromic Pierre Robin Sequence only. The prevalence in our study was lower than the Pierre Robin Sequence prevalence worldwide (0.04/1000 and 0.12/1000 live births, respectively) [37]. The number of Pierre Robin Sequence patients in our research were fewer than the 72 cases reported by Aziza et al. [38] over a seven-year period (2002–2008). This is because our study is a prevalence study conducted on maternity hospitals (30 hospitals). On the other hand, Aziza’s study was a single hospital based study that recruited their sample from a referral center. In addition, our study exceeded the number of cases by 25 as compared to that of Hadadi et al. [35] over a seven-year period (2002–2008).

### Consanguinity

In our study, consanguinity was recorded in 62.9% of NSOFC patients, which is greater as compared to the prevalence reported in the Saudi community by EL-Hazmi et al. [39] (57.7%), Al-Johar et al. [33] (54.4%) and El Mouzan et al. [25] (57%). However, it was comparable to the 65.9% rate of consanguineous marriages among NSOFC patients reported by Sabbagh et al. [9]. Of the total newborns having consanguineous parents, first cousins accounted for 79 patients (72.5%), which was consistent with the reports of El-Hazmi et al. [39] (41%) and Sabbagh et al. [9] (54.3%), for general Saudi population. A meta-analysis that found a significant correlation between NSOFC prevalence and first cousin consanguineous marriages (OR: 1.49; 95% CI; 1.07 to 2.07) showed similar findings [28].

#### COVID-19

Numerous variables, including an increase in cigarette smoking during COVID-19 pandemic by 9.1%, may have contributed to the rise in NSOFC cases during lockdown period [40]. Mothers also had higher levels of psychological anguish, anxiety, and depression during this phase [16]. Last but not least, there may be a connection between COVID-19 infection, symptoms, and the prevalence of NSOFC; however, current investigations have not supported this connection. As a result, this study is the first to document NSOFC prevalence rate, which is greater than that documented prior to the pandemic. Additionally, it is the first study to document the immunization rates among NSOFC mothers. Mothers who were exposed to Covid-19 infection in the first trimester comprised 21 (12.1%) of the overall sample population. In addition, compared to CP and CL, statistically more mothers with CLP children had Covid-19 infection. In contrast, statistically fewer mothers of CLP children received the Covid-19 infection vaccine than did mothers of CL infants. This would suggest that COVID-19 has a propensity to make CL/P more severe. But further research is needed to verify this theory.

This study highlighted some setbacks; the first limitation of this study is the exclusion of the private sector, however since the private sector represent minor percentage of Saudis (20%), this constraint may be overlooked [41]. Second, stillbirths were also excluded from this study that can skew results on NSOFC prevalence. Nevertheless, according to the Saudi Arabian Ministry of Health, just 11 stillbirths per 1000 live births, occurred in 2020 [41]; consequently, this should not deteriorate our study findings.

## Conclusion

In Saudi Arabia, NSOFC incidence was 1.26 per 1,000 live births which is higher than what was reported before the pandemic (1.17/1000 live births), and lower than cumulative worldwide prevalence (1.47/1000 live births). Pierre Robin Sequence was present in 0.04/1000 live births frequency. In addition to reporting Covid-19 infection prevalence and vaccine exposure among NSOFC's mothers, this study represents the first of its type to evaluate NSOFC prevalence in Saudi Arabia on a national level.

## Data Availability

The data that support the findings of this study are available from the corresponding author upon reasonable request.
